# Immunoprofiling of leukemic stem cells CD34+/CD38−/CD123+ delineate FLT3/ITD-positive clones

**DOI:** 10.1186/s13045-016-0292-z

**Published:** 2016-07-27

**Authors:** Adhra Al-Mawali, David Gillis, Ian Lewis

**Affiliations:** 1 Division of Human Immunology and Haematology, SA Pathology, Hanson Institute, Frome Road, Adelaide, SA 5000 Australia; 2 Centre of Studies and Research, Ministry of Health, Muscat, Sultanate of Oman

**Keywords:** Acute myeloid leukemia, FLT3/ITD, Leukemic stem cells, CD34+/CD38−/CD123+, Flow cytometry

## Abstract

**Background:**

Acute myeloid leukemia (AML) is a heterogeneous clonal disorder presenting with accumulation of proliferating undifferentiated blasts. Xenograft transplantation studies have demonstrated a rare population of leukemia-initiating cells called leukemic stem cells (LSCs) capable of propagating leukemia that are enriched in the CD34+/CD38− fraction. LSCs are quiescent, resistant to chemotherapy and likely responsible for relapse and therefore represent an ideal target for effective therapy. LSCs are reported to overexpress the alpha subunit of the IL-3 receptor (CD123) compared to normal CD34+/CD38− hematopoietic stem cells. It has not been demonstrated whether CD123-positive (CD34+/CD38−) subpopulation is enriched for any clonal markers of AML or any LSC properties. The aims of this study were to investigate whether FMS-like tyrosine kinase (FLT3)/internal tandem duplication (ITD) mutations are present at LSC level and whether FLT3/ITD mutation is confined to LSC as defined by CD34+/CD38−/CD123+ and not CD34+/CD38−/CD123−.

**Methods:**

Thirty-four AML cases were analyzed by five-color flow cytometry and sequential gating strategy to characterize of CD34+/CD38−/CD123+ cells. These cells were sorted, analyzed by PCR, and sequenced for FLT3/ITD.

**Results:**

In this study, we confirm significant expression of CD123 in 32/34 cases in the total blast population (median expression = 86 %). CD123 was also expressed in the CD34+/CD38− cells (96 ± 2 % positive) from 28/32 for CD123+ AML. CD123 was not expressed/low in normal bone marrow CD34+/CD38− cells (median expression = 0 %, range (0–.004 %). AML samples were tested for FLT3/ITD (10 positive/25). FLT3/ITD+ AML cases were sorted into two putative LSC populations according to the expression of CD123 and analyzed for FLT3/ITD again in the stem cell fractions CD34+/CD38−/CD123+ and CD34+/CD38−/CD123−. Interestingly, FLT3/ITD was only detected in CD34+/CD38−/CD123+ (7/7) and not in CD34+/CD38−/CD123− subpopulation (6/7).

**Conclusions:**

This finding shows that FLT3/ITD are present at LSC level and may be a primary and not secondary event in leukemogenesis, and the oncogenic events of FLT3/ITD happen at a cell stage possessing CD123. It shows that CD123 immunoprofiling provides further delineation of FLT3+ LSC clone. This novel finding provides a rationale for treatment involving CD123-targeting antibodies with intracellular FLT3 inhibitors directed against CD34+/CD38−/CD123+. This may result in more effective anti-LSC eradication.

## Background

Acute myeloid leukemia (AML) represents a group of clonal hematopoietic stem cell disorders in which both failure to differentiate and increased proliferation potential in the stem cell compartment result in accumulation of non-functional cells termed myeloblasts. While the majority of patients with AML achieve a complete remission (CR) with induction therapy, more than half of these subsequently relapse and eventually die of the disease [1]. Relapse is thought to occur because of the failure of chemotherapy to eradicate non-proliferating but self-renewing leukemia stem cells [2]. Human AML stem cells, the so-called leukemic stem cells (LSCs), have been defined as CD34+/CD38− cells with severe combined immunodeficient (SCID) mouse-repopulating ability, which is a reflection of their capacity to self-renew [3, 4].

Previous studies suggest that LSCs are biologically distinct from more mature leukemic blasts and may not be responsive to conventional chemotherapeutic regimens [5–7]. One potential difference between normal and leukemic cells lies in their response to hematopoietic growth factors. It could be speculated that differential sensitivity to cytokines may also exist at the stem cell level [8]. A potential unique marker of LSCs is interleukin-3 receptor alpha chain (CD123) which has been shown to be highly expressed on leukemic but not normal CD34+/CD38− hematopoietic cells, with a negative impact on the outcome and prognosis in AML patients [8–10] (Table 1).

**Table 1:** Clinical characteristics of AML patients according to FLT3/ITD status

Patient characteristics	AML pos for FLT3/ITD	AML neg for FLT3/ITD
No. of patients	12	27
Male/female	8/4	17/10
Age at diagnosis, median, (range)	62 (23–73)	69 (23–88)
WBC count at diagnosis × 10^9^/L, median (range)	6.6 (0.9–179)	6.3 (0.7–227)
BM blasts % by morphology, median (range)	72 (26–85)	35 (20–87)
AML de novo/secondary *n* (%)	9 (75)/3 (25)	23 (85)/4 (15)
FAB classification, *n* (%)		
Mo	0 (0)	0 (0)
M1	6 (50)	2 (7)
M2	1 (8)	10 (37)
M3	2 (17)	2 (7)
M4	1 (8)	3 (11)
M5	1 (8)	4 (15)
M6	0 (0)	0 (0)
M7	0 (0)	0 (0)
Not classified	1 (8)	6 (22)
Cytogenetic risk group, *n* (%)		
*Favorable*	2 (17)	4 (15)
Intermediate	10 (83)	13 (48)
Poor	0 (0)	9 (33)
Insufficient sample	0 (0)	1 (4)
Induction therapy response, *n* (%)		
CR	7 (70)	15 (88)
Failure	3 (30)	2 (12)

FMS-like tyrosine kinase (FLT3), which belongs to a group of class III receptor tyrosine kinases, is preferentially expressed on hematopoietic stem/progenitor cells and plays a role in both differentiation and proliferation [11, 12]. FLT3 is also expressed on the leukemic blasts in the majority of cases of acute leukemia, even in CD34-negative cases [13–15]. Internal tandem duplications (ITDs) of varying length in the juxta-membrane (JM) region occur due to constitutive activation of the FLT3 receptor and are correlated with poor prognosis in AML patients [16–18].

The aim of this study was to investigate whether or not FLT3/ITD mutations are present at LSC level. We explore whether or not FLT3/ITD mutation is confined to the population of LSC as defined by CD34+/CD38−/CD123+.

Therefore, we explored the issue of whether or not FLT3/ITD mutations are present at LSC level as defined by the phenotype CD34+/CD38−/CD123+. Seven primary AML samples harboring FLT3/ITD mutations were sorted into stem cell-enriched fractions CD34+/CD38−/CD123+ and stem cell-enriched fractions lacking CD123, and FLT3/ITD were then analyzed in the two-sorted fractions. Our data provide the first definitive evidence that FLT3/ITD mutations occur at LSC level at a stage of cells that possess interleukin-3 (IL-3) α receptor (CD123). It is speculated that FLT3/ITD mutation could make the LSCs more capable of expanding in the environment and development of leukemia [19].

## Methods

### Patients

The clinical characteristics of AML patients with FLT3/ITD mutation and FLT3/ITD wild type and correlation with different FAB subtypes are demonstrated in Table 1.

Thirty-four consecutive, unselected, newly diagnosed, and untreated AML adult patients were analyzed at diagnosis for the expression of CD123 in the total blast population and at stem cell level as defined by CD34+/CD38−. Diagnoses were established according to criteria proposed by the French-American-British (FAB) study group [20]. The patients’ characteristics are shown in Table 2.

**Table 2:** Patient characteristics

Patient characteristics	Total (%)
No. of patients	34
Male/female	24/10
Age at diagnosis, mean (range)	63 (23–86)
% blasts at diagnosis (morphology), mean (range)	41.5 (20–96)
% blasts at diagnosis (flow), mean (range)	42.5 (9–86)
WBC count at diagnosis, 10^9^/L, median (range)	4.5 (0.71–179)
De novo/secondary AML	27 (79)/7 (21)
FAB classification, *n* (%)	
M_0_	0 (0)
M_1_	8 (24)
M_2_	10 (29)
M_3_	1 (3)
M_4_	2 (6)
M_5_	4 (12)
M_6_	1 (3)
M_7_	0 (0)
Not classified	8 (24)
Cytogenetic risk group, *n* (%)	
Favorable	2 (6)
Intermediate	19 (56)
Poor	12 (35)
No metaphases	1 (3)
FLT3/ITD, *n* (%)	
Present	10 (29)
Absent	15 (44)
Not analyzed	9 (26)
CD123, *n* (%)	
Present	32 (94 %)
Absent	2 (6 %)

### Controls

For control purposes, we examined normal bone marrow (BM) cells obtained from five healthy volunteers. All controls were treated in the same manner as patient samples.

### Study conduct

All patients and controls gave their informed consent for participation in the current evaluation after having been advised about the purpose and investigational nature of the study as well as potential risks. The study design was approved by the Research Ethics Committee of the Royal Adelaide Hospital, South Australia prior to its initiation.

### Monoclonal antibodies

A number of commercial monoclonal antibodies (MoAbs) (against CD13, CD33, CD38, CD123, CD45, CD34, CD117, and HLA-DR) were used to characterize and isolate leukemic stem cells. A list of MoAbs is shown in Table 3. To determine expression of CD antigens on blasts and leukemic stem cells, combinations of CD13/CD33/CD38/CD123/CD45/CD34/CD117/HLA-DR were applied.

**Table 3:** Specification of monoclonal antibodies

Antibody	Clone	Isotype	Conjugate	Source
CD13	L138	IgG1	PE	Beckton Dickinson
CD33	P67.6	IgG1	PE	Beckton Dickinson
CD33	P67.6	IgG1	FITC	Beckton Dickinson
CD34	8G12	IgG1	FITC	Beckton Dickinson
CD34	581	IgG1	PC5	ImmunoTech
CD38	LS198-4-3	IgG1	PE	Beckman Coulter
CD45	Immu19.2	IgG1	PC5	ImmunoTech
CD45	J33	IgG1	ECD	ImmunoTech
CD117	104D2D1	IgG1	PE	ImmunoTech
CD117	104D2D1	IgG1	PC7	Beckman Coulter
CD123	9F5	IgG1	PE	Beckton Dickinson
HLA-DR	L243	IgG2a	FITC	BioDesign
CD38	T16	IgG1	FITC	Beckman Coulter

### Five-color multiparameter flow cytometry and characterization of CD34+/CD38−/CD123+ cells

Heparinized bone marrow cells (~106/tube) were incubated with combinations of MoAbs at room temperature for 15 min. Erythrocytes were then lysed in 2 mL FACSTM lysing solution (Becton Dickinson, San Diego, CA, USA). Cells were consequently washed and analyzed on a Cytomics® FC500 flow cytometer and CytomicsTM CXP Analysis Version 1 Software (Beckman Coulter, USA).

Leukemic progenitors were defined by their phenotype (CD34+, CD45+, CD38−, CD123+) using CD45 gating strategy [21] and CD34 and/or CD117 backgating strategy to better define the blast population as previously described by our group [22]. Control tubes stained with an isotype-matched control were included in all experiments and were used to define the cutoff point for positive/negative staining.

The gating strategy in newly diagnosed AML to identify CD34+/CD38−/CD123+ cells is as shown in Fig. 2. After labeling of AML cells with the appropriate antibody combinations, the CD34+/CD38− cells were identified by a CD45 dim/SS low strategy based on CD34 backgating (Fig. 2a), gating on blasts characterized by CD45 dim/low side scatter (SSC) (Fig. 2b), and gating of the blasts within the gate defined by forward scatter (FSC) and SSC to identify a population that is roughly homogeneous for scatter properties (Fig. 2c). The CD38−, CD34+ and CD123+ population were defined using isotype matching as a negative control (Fig. 2d, e). Cells from the FSC/SSC plot defined in Fig. 2c are shown in Fig. 2g, h in a plot defined by CD34 and CD38 expression. The CD34+/CD38− population defined in Fig. 2g, h is gated in a FSC/SSC plot to identify a CD34+/CD38− population with homogeneous scatter properties (Fig. 2i). The frequency of this determined CD34+/CD38− population was used in this study. Cells from the FSC/SSC plot defined in Fig. 2i are shown in Fig. 2j, k in a plot defined by CD34 and CD123 expression. The CD34+/CD38−/CD123+ population defined in Fig. 2j, k is gated in a FSC/SSC plot to identify a CD34+/CD38−/CD123+ population with homogeneous scatter properties Fig. 2l.

The gating strategy in normal BM to identify CD34+/CD38−/CD123+ is as shown in Fig. 3; after labeling of normal BM cells with the appropriate antibody combinations, the CD34+ cells were identified by a CD45 dim/SS low and CD34+ backgating strategy (Fig. 3a–c). Gating on CD34+ cells and blasts characterized by CD45 dim/low SSC showing in red (Fig. 3c). The CD38+ and CD123+ population were defined using isotype matching as a negative control and backgating of the positive cells for CD123 (Fig. 3d, e) and CD38 (Fig. 3g, h) in total population. The positive cells for CD123 are showing in blue (Fig. 3f) and in pink (Fig. 3i) for CD38 in CD45/SSlog dot plot. Cells from the plot defined in Fig. 3b are shown in Fig. 3j in a plot defined by CD34 and CD38 expression. The frequency of the CD34+/CD38− cells determined CD34+/CD38− population was used in this study. The frequency CD34+/CD38−/CD123+ and CD34+/CD38−/CD123− population was defined in Fig. 3k in a plot defined by CD34 and CD123.

### Isolation of CD34+ cells

The isolation of CD34+ cells was performed on mononuclear cells (MNC) from the BM of 34 AML patients. MNC were washed twice with MACS CD34+ buffer, and CD34+ progenitor cells were purified using a MACS CD34+ progenitor cell selection isolation kit (Miltenlyi Biotech, Germany) according to the manufacturer’s instructions. The purity of CD34+ cells following the isolation procedure was calculated by staining with an anti-CD34-PC5.

### Sorting of leukemic stem cells CD34+/CD38−/CD123+

We sorted the CD34+/CD38− population into the putative LSC population CD34+/CD38−/CD123+ and the putative normal hematopoietic stem cell (HSC) population CD34+/CD38−/CD123− using BD FACSARIA™ Cell Sorter (BD Biosciences, San Jose, CA 95,131, USA). Cells were incubated with a phycoerythrin (PE)-conjugated CD123 MoAb, a fluorescein isothiocyanate (FITC) CD38 MoAb, and a phycoerythrin-Cy5 (PC5)-conjugated CD34 MoAb in AB serum at room temperature for 15 min. Then, cells were washed, and the CD34+/CD38−/CD123+ fraction of cells isolated in a BD FACSARIATM Cell Sorter. Pools of cells expressing similar level of CD34+/CD38−/CD123+ and CD34+/CD38−/CD123- were collected. After sorting, the purity of CD34+/CD38−/CD123+ AML cells was >95 %.

### Polymerase chain reaction for FLT3

We isolated genomic deoxyribonucleic acid (DNA) from the purified sorted population using a QIAamp Mini Kit (QIAGEN) and polymerase chain reaction (PCR) was performed using primers flanking the (JM) coding region: 11F (GCAATTTAGGTATGAAAGCCAGC) and 12R (CTTTCAGCATTTTGACGGCAACC) previously described by Nakao et al. [16]. The amplification was performed on DNA Thermal Cycler (Eppendorf Mastercycler) and entailed an initial denaturation of 94 °C for 7 min, followed by 35 cycles of denaturation at 94 °C for 1 min, annealing at 62 °C for 1 min, and extension at 72 °C for 1 min, with a final extension at 72 °C for 7 min. PCR products were resolved on 2 % agarose gels and visualized under ultraviolet light after ethidium bromide staining. The characteristic doublet of the FTL3/ITD mutation is easily visualized after electrophoresis. Genomic DNA from known positive and negative cases was used as controls.

### Optimization of the PCR technique for low DNA concentrations

**Fig. 1:**
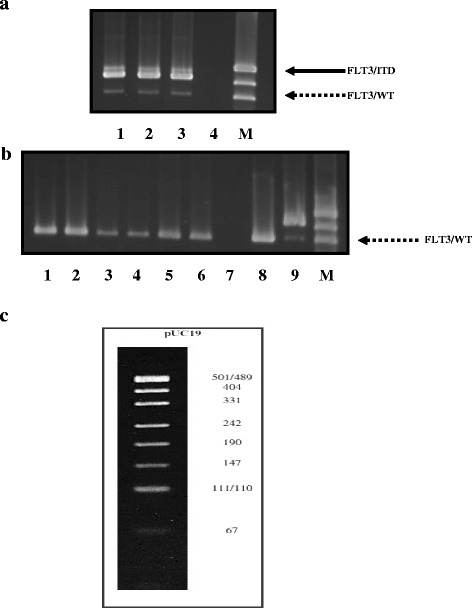
Optimization of PCR technique using FLT3/ITD-positive and FLT3/ITD-negative patient. **a** Using three different DNA concentrations 5, 2.5, and 1.25 ng/μl in FLT3/ITD-positive AML patient. *Lane 1* FLT3/ITD detected with 50 ng DNA added, *lane 2* FLT3/ITD detected with 25 ng DNA added, *lane 3* FLT3/ITD detected with 12.5 ng DNA added, *lane 4* no DNA added, and *M* pUC19 molecular marker. The *solid line* points to FLT3/ITD bp inserted while the *dotted arrow* points to the WT FLT3 gene. **b** Using DNA obtained from different number of cells (2 × 10^6^, 150, 1606 cells in duplicates) on WT FLT3 AML patient based on the number of cells obtained from the first sorted sample (sample no.1 in Table 4). *Lanes 1 and 2* (duplicate) DNA obtained from 2 × 10^6^ cells, FLT3 WT detected; *lanes 3 and 4* (duplicate) DNA obtained from 150 cells, FLT3 WT detected; lanes 5 and 6 (duplicate) DNA obtained from 1606 cells, FLT3 WT detected; lane 7, no DNA, so the PCR was specific; lanes 8 and 9 are the negative and positive controls and *M* was pUC19 marker. **c** DNA fragments (bp) of the molecular marker pUC19

To optimize the PCR method for very low DNA concentrations obtained from very few numbers of cells, a study was conducted using different DNA amounts 50, 25, and 12.5 ng with 15 μl PCR reaction mix, in a total volume of 25 μl in duplicate from an AML patient positive for FLT3/ITD. The results of this experiment revealed that FLT3/ITD could be detected at 5, 2.5, and 1.25 ng/μl DNA as shown in Fig. 1a. Thus, we used 12.5 ng DNA in sorting experiments for PCR.

### Sequencing

Two of the ITD mutations identified during the assay validation were cycle sequenced in the forward and reverse direction to verify the results. PCR products were purified using QIAQuick columns (QIAGEN) and cycle sequenced using Big Dye, Version 2 (Applied Biosystems) according to the manufacturer’s protocol. For sequencing the ITD, PCR primers 11F (5′-GCAATTTAGGTATGAAAGCCAGC-3′) and 12R (5′-CTTTCAGCATTTTGACGGCAACC-3′) of exons 14 and 15 were used. Sequences were aligned and examined using Mutation SurveyorTM software.

## Results

### Expression of CD123 (IL-3 α receptor) in AML blast cells

 Thirty-four AML patients at diagnosis were tested for the expression of CD123 in the total blast population and at the stem cell level as defined by CD34+/CD38−.

CD123 was expressed in 32 of 34 (94 %) AML patients. The median expression in the whole blast population was 86 % (range, 20–99 %). In 24 (75 %) patients, the majority of blasts (>60 %) expressed CD123 and in the remaining 8 (25 %) patients, only a subset of blasts expressed CD123 (Table 2).

### Expression of CD123 (IL-3 α receptor) in AML stem cells CD34+/CD38−

**Fig. 2:**
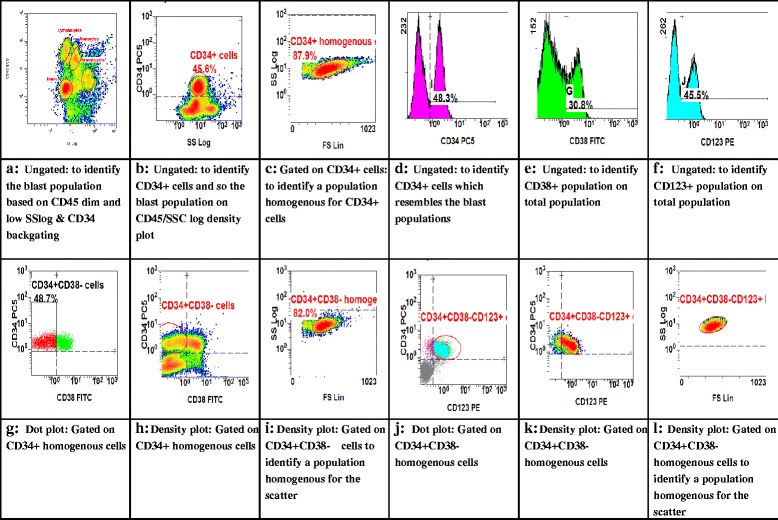
Gating strategy in newly diagnosed AML to identify CD34+/CD38−/CD123+ cells

The expression of CD123 on the stem cell fraction as defined by CD34+/CD38− was tested using CD45 and CD34 backgating strategy outlined in Fig. 2. Four patients were CD34 negative, and therefore, the estimation of CD123 expression in the CD34+/CD38− compartment was not possible. Two of these patients were M5a (generally most of M5a patients are CD34 negative), one M1 and one M3 (in most cases, M3 are also CD34 negative) FAB classification. CD123 was strongly expressed in the CD34+/CD38− cells (96 ± 2 % positive) from 28 (87.5 %) of 32 primary specimens.

### Expression of CD123 (IL-3 α receptor) in normal BM CD34+/CD38− fraction

**Fig. 3:**
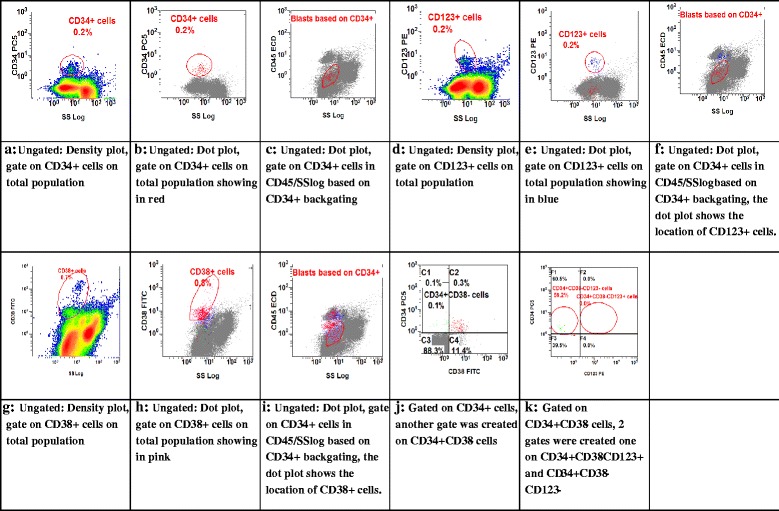
Gating strategy in normal BM to identify CD34+/CD38−/CD123+ cells

Five normal BMs were tested for the expression of CD123 on CD34+/CD38− cells, and they were all CD123 negative. The median level of CD123 in normal CD34+/CD38− stem cells (0.119 %), range (0.004–1.43 %) in the five normal BMs (Fig. 3).

### Sorting AML stem cells

We analyzed FLT3 mutation status in 25 of 32 (78 %) patients who expressed CD123, 10 were FLT3/ITD positive and 15 were wild type (WT).

**Fig. 4:**
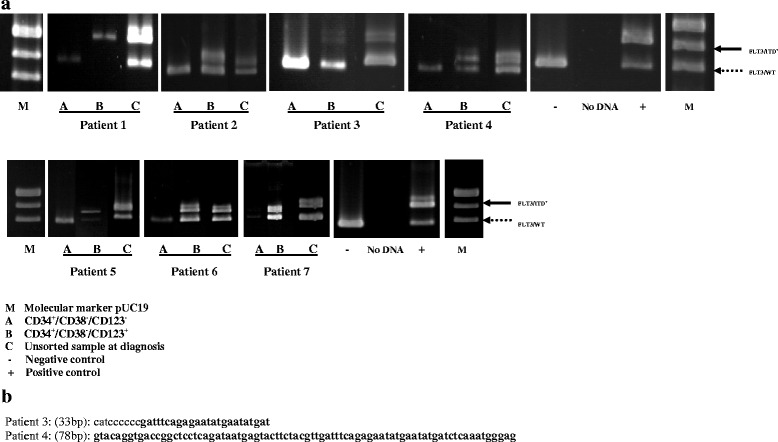
FLT3/ITD mutations in the BM cells of 7 AML patients. **a** CD34+/CD38−/CD123+ and CD34+/CD38−/CD123− cells from different patient samples were isolated by sorting (sorting results displayed in Table 4). For each of the seven samples, genomic DNA was isolated from the (*1*) CD34+/CD38−/CD123−, (*2*) CD34+/CD38−/CD123+ cells, and (*3*) unsorted cells at diagnosis (a *vertical line* was inserted to indicate that diagnosis samples were done on separate gels). **b** The sequences of the FLT3/ITD mutations from the two samples (patients 3 and 4) are shown. These sequences, obtained from the DNA of CD34+/CD38−/CD123+ cells, matched the sequences of the mutations present in the pre-sorted samples. *bp* indicates base pair. *Boldface sequence* refers to the inserted, duplicated material

To determine the expression of FLT3/ITD in AML stem cells, highly purified (purity >95 %) CD34+/CD38−/CD123+ and CD34+/CD38−/CD123− cells were examined for FLT3/ITD mutation in seven patients with FLT3/ITD-positive AML as demonstrated in Fig. 4. We were unable to perform analysis in the remaining three FLT3/ITD-positive patients because of insufficient material. The numbers of CD34+/CD38−/CD123+ cells sorted ranged from 150 to 300,000 cells, and 16 to 148,396 cells for CD34+/CD38−/CD123− cells (Table 4). Immunomagnetic cell selection (MACS) was used to enrich CD34 cells from patient no. 4, as CD34 in this patient was only 2.3 % (Table 4).

**Table 4:** Summary of results from sorting and immunomagnetic cell selection MACS

Sample no.	Age	Total unsorted cells	% CD34 unsorted cells	% CD123 unsorted cells	Total CD34+/CD38−/CD123+ cells from the sorter	Total CD34+/CD38−/CD123− cells from the sorter	WBC count ×10^9^/L	FAB-subtype	Cytogenetics	% blast % diagnosis	Sequencing FLT3/ITD
1	46	10 × 10^6^	96	86	150	1606	5.6	M1	Normal	80	ND
2	60	18 × 10^6^	89	99	76,000	418	4.5	M1	Normal	62	ND
3	69	12 × 10^6^	91	86	12,674	148,396	14.4	M2	Normal	22	78 bp
4	53	27 × 10^6^	73^a^	93	1839	16	179	M5a	Normal	82	33 bp
5	62	30 × 10^6^	10	64	559	164	145	M4	Normal	40	ND
6	65	17 × 10^6^	15	99	300,000	69	6.11	M2	Normal	73	ND
7	72	40 × 10^6^	79	68	640	3602	0.92	M1	Normal	26	ND

Total unsorted cells represents the starting sample size, whereas total CD34+CD38−CD123+ and CD34+CD38−CD123− cells refers to the total number of CD34+/CD38−/CD123+/− cells obtained from a given sample after sorting. Genomic DNA was sequenced for FLT3/ITD for sample nos. 3 and 4. *ND* indicates not done

^a^After CD34+ selection by MACS, the total yield of CD34+ = 2.4 × 10^−5^ cells

All of CD34+/CD38−/CD123+ fractions amounted to less than 1 % of the total cells except sample number 6. In addition, with the exception of sample number 3, all of CD34+/CD38−/CD123− fractions also amounted to less than 1 % of the total cells (Table 4).

### Detection of FLT3/ITD in the sorted AML stem cells

Genomic DNA from unsorted and sorted cells was isolated and PCR was performed using primers flanking exons 14 and 15 of the *FLT3* gene [23, 24]. In the seven positive FLT3/ITD samples analyzed, the mutation was detected in the LSC-enriched fraction CD34+/CD38−/CD123+ (Fig. 4a). In six patients, CD34+/CD38−/CD123− cells were FLT3/ITD negative. In the remaining patient, it is possible that no true CD123− cells were obtained as CD123 was expressed on 99.2 % of CD34+/CD38− cells and very low numbers of cells were collected in CD34+/CD38−/CD123− fraction (69 cells only).

In sample 1, the mutation in LSC-enriched fraction CD34+/CD38−/CD123+ was most likely present in homozygous form, as less than 1 % of the PCR product represented the WT *FLT3* gene. In samples 2, 3, 4, 5, 6, and 7, the mutations were present in heterozygous form, as approximately 50 % of the PCR product represented the WT *FLT3* gene, demonstrated by thickness of the band (Fig. 4a).

### Sequencing the FLT3/ITD

The FLT3/ITD mutations in the CD34+/CD38−/CD123+ cells were sequenced in two patients (patients 3 and 4) (Fig. 4b) to confirm that they represented the identical mutations present in the sorted and unsorted original samples. In one patient, 33 base pairs (bp) inserted, and the other one was 78 bp inserted. The fact that this expanded population of LSCs contained the identical FLT3/ITD mutation as was observed in the original unsorted samples constitutes evidence that the FLT3/ITD mutations were present in LSCs.

## Discussion

In this study, we hypothesized that the FLT3/ITD mutation occurs at a stage of stem cells defined by CD34+/CD38− and IL-3 α receptor in AML patients, and FLT3/ITD may be a primary and not a secondary event in leukemogenesis.

We have identified that expression of CD123 is found on virtually almost all (94 %) AML specimens examined similar to previously published data [8, 25, 26]. The high levels of expression observed could simply be indicative of some other conserved event in leukemogenesis. Furthermore, CD123 expression was also demonstrated on the primitive subpopulation of CD34+/CD38− cells (28 of 32 specimens).

The presence of CD123 on AML CD34+/CD38− cells has a potential significance. It demonstrates that LSCs are biologically distinct from their normal stem cell counterparts. In addition, because CD123 is not found on normal HSCs, it provides a unique marker that can be used to identify the malignant clone. This feature may be very useful in minimal residual disease studies as a single and standardized marker [27–29]. Furthermore, the CD123 epitope represents a target to which therapeutic strategies may be directed [8, 25, 26, 30–32].

Somatic mutation of FLT3 involving ITDs of the JM domain have been identified in approximately 17–34 % of AML cases [16, 17, 33–37]. Two studies demonstrated the presence of FLT3 mutations at LSC level [37, 38]. Levis et al. [38] sorted primary AML samples harboring FLT3/ITD mutations into stem cell-enriched CD34+/CD38− fractions and then analyzed the sorted and unsorted cells for the FLT3 mutant-WT ratio. In each case, the FLT3 mutant-WT ratio was not changed by selection of CD34+/CD38− cells, implying that the mutations occurred in the LSCs. The stem cell-enriched fraction engrafted non-obese diabetic-severe combined immunodeficient (NOD/SCID) mice, and the FLT3/ITD mutation was present in the resultant engrafted marrow. In addition, the finding that BM cells from patients with AML harboring FLT3/ITD mutations had a greater capacity to engraft NOD/SCID mice than cells from patients lacking such mutations also supports the hypothesis that FLT3/ITD is present at LSC level and hence more likely to engraft the NOD/SCID mice [39, 40].

We demonstrate that FLT3/ITD mutations are found in a primitive fraction of cells as defined by CD34+/CD38−/CD123+. In addition, our data show that the FLT3/ITD mutations were present within purified enriched LSCs defined by CD123 and absent within stem cells without CD123. The specimens were derived from several different FAB subtypes M1, M2, M4, and M5a, and therefore, represent a broad cross section of commonly detected AML types.

The sorted cells in most of the samples comprised less than 1 % of the total population before sorting. If CD34+/CD38−/CD123+ subset contains a significant fraction of LSCs, then the FLT3/ITD imply that most of these mutations are in cells capable of self-renewal [19]. Ideally, these enriched leukemic cells should be injected into NOD/SCID mice but this was technically challenging as an appropriate AML model was not available in the country.

In sample 1, after sorting of the CD34+/CD38−/CD123+ fraction, no WT signal was detectable, probably as a result of eliminating the small percent of normal hematopoietic cells in the sample. In all other six cases, the FLT3/ITD was very similar between sorted and unsorted fractions as shown in Fig. 4. In no case was the mutant allele depleted or enhanced by sorting for LSCs. It is interesting to note that the six samples were heterozygous for FLT3/ITD, even in the stem cell-sorted fractions. It is possible that this reflects a PCR bias for the shorter WT molecule and that this ratio actually represents 100 % of cells with a heterozygous mutation. Alternately, these ratios may suggest the presence of either additional sub clones of leukemic CD34+/CD38−/CD123+ cells that lacked the mutation within these samples.

FLT3-ITD mutation may occur in the early phase of leukemia pathogenesis, even blast cell with FLT3-ITD mutation are eliminated by chemotherapy, the aberrant mutation that pre-existed in LSC could still play an indispensable role in the relapse of the leukemia [41–43], and our data provided new insights into the pathogenesis of LSC.

Several lines of evidence have previously suggested that these mutations can occur relatively late in the development of leukemia. Few studies have demonstrated that in a small proportion of cases, FLT3/ITD mutations are lost at relapse [44–48]. This suggests that, in these few cases, the mutations occurred in a subclone that was eliminated by treatment. Until now, the observation of an increased outgrowth of FLT3/ITD AML in NOD/SCID mice suggests that in a large part of the FLT3/ITD-positive leukemia, the mutation is present at the level of the malignant stem cell [19, 49]. Nevertheless, it is not clear if the observed outgrowth is solely dependent on the transplanted LSCs or that also other, more committed, cells are able to expand in this model. The occurrence of an enhanced survival of FLT3/ITD AML cells is further supported by the observations of Schnitgger et al. [33] and Kottaridis et al. [45] who observed that FLT3/ITD patients who relapsed for the greater part showed an increased mutant to WT ratio at relapse.

Our results are not consistent with the previous findings of occasional loss of FLT3/ITD mutations at relapse. However, our results do not exclude that at least a subset of AML, the FLT3/ITD mutations are present only in subclones derived from the original LSC, subclones that lack the capacity for self-renewal. It may be that our sample size was simply not large enough to uncover such cases. These may represent the cases in which the mutation arose as a relatively late hit in leukemogenesis and may be the cases in which the mutation is lost at relapse. Alternately, FLT3 mutations could always be present in LSCs, but occasionally chemotherapy succeeds in eradicating the FLT3/ITD samples, whereas other LSCs that lack the mutation are resistant.

In the study of Masao et al. [50], they identified target genes of the FLT3/ITD by microarray expression profiling. ITD mutations induced transcriptional programs that partially mimicked IL-3 activity with many genes being specifically regulated by the mutations but not by ligand-activated WT FLT3. They also have shown that FLT3/ITD mutations induce a transcriptional program that is fundamentally different from the program induced by FLT3-WT.

The sorting strategy used in this study implemented stringent criteria for scoring, as the purity of sorting was >95 % in all the seven AML patients. We also optimized the PCR for very small number of cells and were able to demonstrate the presence of FLT3/ITD in CD34+/CD38−/CD123+ fraction and not CD34+/CD38−/CD123−. This confirms that the PCR technique was robust (with the exception of one patient) which gives evidence that the technique is working on small number of cells.

Targeting of CD123/IL-3 alpha receptor may be a novel promising treatment approach in patients with CD123+ AML [8, 25, 27, 51–53]. This concept is based on the notion that in most patients with AML, myeloblasts express CD123 as shown in our study.

## Conclusions

In conclusion, these novel findings show that FLT3/ITD mutations are present at the leukemic stem cell level and may be a primary and not secondary event in leukemogenesis. There is also evidence to suggest that FLT3/ITD mutations were *present* within purified enriched leukemic stem cells defined by CD123 (CD34+CD38−CD123+) and *absent* within stem cells without CD123 (CD34+CD38−CD123−). Furthermore, the study shows that the oncogenic events of FLT3/ITD happen at a cell stage possessing the alpha chain of the IL-3 receptor (CD123). These novel findings provide a rationale for treatment involving CD123-trageting antibodies with intracellular FLT3 inhibitors directed against AML stem cells.

In the future, it would be of great interest to study the frequency of CD34+/CD38−CD123+ cells post induction and consolidation chemotherapy as these cells may represent the highly resistant cells to chemotherapy and might be the cells that cause regrowth of leukemia and thus relapse of the disease.

## Abbreviations

AML, acute myeloid leukemia; BM, bone marrow; CR, complete remission; DNA, deoxyribonucleic acid; FAB, French-American-British; FITC, fluorescein isothiocyanate; FLT3, FMS-like tyrosine kinase 3; FSC, forward scatter; IL-3, interleukin-3; ITD, internal tandem duplication; LSC, leukemic stem cell; MNC, mononuclear cells; MoAbs, monoclonal antibodies; NOD/SCID, non-obese diabetic/severe combined immunodeficiency; PC5, phycoerythrin cyanin 5; PCR, polymerase chain reaction; PE, phycoerythrin; SCID, severe combined immunodeficient; SSC, side scatter
